# The Antibacterial Activity of Dayak Onion (*Eleutherine palmifolia* (L.) Merr) towards Pathogenic Bacteria

**DOI:** 10.21315/tlsr2018.29.2.4

**Published:** 2018-07-06

**Authors:** Tiara Dini Harlita, Oedjijono, Ari Asnani

**Affiliations:** 1 Department of Biology, Faculty of Biology, Universitas Jenderal Soedirman, Jl. Dr. Soeparno, Karangwangkal 53123, Purwokerto, Jawa Tengah, Indonesia; 2 Department of Health Analyst, Politeknik Kesehatan Kemenkes Kaltim, Jl. Kurnia Makmur No. 64 Kel. Harapan Baru, Samarinda, Kalimantan Timur, Indonesia; 3 Department of Chemistry, Faculty of Mathematic and Natural Sciences, Universitas Jenderal Soedirman, Jl. Dr. Soeparno, Karangwangkal 53123, Purwokerto, Jawa Tengah, Indonesia

**Keywords:** Antibacterial, Bioautography, Inhibition Activity, Dayak Onion

## Abstract

Antibacterial activity of indigenous Dayak onion (*Eleutherine palmifolia* (L.) Merr) was investigated. The Dayak onion was solvent extracted with *n*-hexane, ethyl acetate, and ethanol 96% consecutively. Each extract was tested its antibacterial activity towards methicillin-resistant *Staphylococcus aureus* (MRSA), *Bacillus cereus*, *Shigella* sp., and *Pseudomonas aeruginosa* using disc diffusion method. The test results showed that the *n*-hexane, ethyl acetate, and ethanol 96% extracts positively inhibited the growth of MRSA, *B. cereus*, *Shigella* sp., and *P. aeruginosa*. The highest inhibition activity of each extract was obtained with 10 mg/mL of extract concentration; whereas the minimum inhibitory concentration (MIC) of each extract was 2 mg/mL. Extract with the highest inhibition activity was ethyl acetate extract against *B. cereus* (139.58%). TLC evaluation of ethyl acetate extract showed four spots and bioautography indicated that ethyl acetate extract contained four types of compounds with inhibition activity against *B. cereus*, in which two compounds have higher antibacterial activity than the other two.

## INTRODUCTION

Dayak onion (*Eleutherine palmifolia* (L.) Merr) is an indigenous plant from Kalimantan Indonesia, which has been used for generations as folk medicine in Dayak community. Empirically, Dayak onion has been used by local people to cure various types of diseases such as high blood pressure, high cholesterol, diabetes, ulcers, constipation, stroke and also as an herbal drink for postpartum mothers. The main part of Dayak onion widely used is its bulb in the form of fresh, dried onion bulb, pickles, or powder (Galingging 2009). Indeed, the potency of Dayak onion as natural multi-function medicine has widespread healing practices. Recently, Couto *et al.* (2016) published an excellent review of *Eleutherine bulbous* as folk medicine. Thus, it is essential to explore Dayak onion for more therapeutic benefits.

The healing effects of Dayak onion are probably due to its active compounds. The bulb of Dayak onion has been reported to have several bioactivities, including antiacne (Syamsul *et al.* 2015), antimitotic (Efendi *et al.* 2015), antifungal (Diana *et al.* 2014), and also antioxidants (Nur 2011; Kuntorini *et al.* 2016). Phytochemical screening on ethanol extract of Dayak onion has indicated that it contained secondary metabolites such as flavonoids, naphthoquinones, anthraquinones, alkaloids, saponins, tannins, triterpenoid and steroid (Kuntorini & Laurentius 2010; Mierza *et al.* 2011).

Antimicrobial activity of Dayak onion has also been reported. Puspadewi *et al.* (2013) have investigated the effectiveness of ethanol extract in inhibiting the growth of bacteria *Staphylococcus aureus*. Different studies showed that the ethanol extract from the bulb of Dayak onion was able to inhibit the growth of *Streptococcus pyogenes* (Kamillah 2014), *Salmonella typhi* (Naafi’ah 2014), and *Escherichia coli* (Amanda 2014). However, those studies mainly used ethanol extract from the bulb. Since not all bioactive compounds which have antibacterial activities dissolve in a polar solvent such as ethanol, thus it is necessary to explore the extraction method using different solvents polarities. Selection of different polarity of solvents is expected to obtain the best solvent for the extraction of antibacterial compounds from the Dayak onion.

Hence, the purpose of this research was to extract the antibacterial compounds from Dayak onion with different polarity of solvents which were non-polar (*n*-hexane), semi-polar (ethyl acetate), and polar (ethanol 96%); to determine the antibacterial activity of all extracts towards pathogenic bacteria namely methicillin-resistant *Staphylococcus aureus* (MRSA), *Bacillus cereus*, *Shigella* sp. and *Pseudomonas aeruginosa*; and to determine the minimal inhibitory concentration (MIC) of all extracts.

## MATERIALS AND METHODS

### Plant Identification

Dayak onion plant was collected from Samarinda, East Kalimantan Indonesia in 2016. The specimen of the plant was identified and authenticated by Dr. Pudji Widodo, MSc from the Laboratory of Plant Taxonomy, Faculty of Biology, Universitas Jenderal Soedirman Purwokerto, Indonesia.

### Sample Preparation

The sample was prepared following Galingging (2009). The whole plant of Dayak onion was washed thoroughly with tap water. The leaves were trimmed off; the bulbs were thinly sliced about ±1–2 mm of thickness and then dried in an oven at 50°C for several hours until constant weight. The dried onion was ground into a powder form, and filtered with 60 mesh sieve. The fine powder obtained was then weighed, sealed, and stored in a dry place.

### Extraction of Antibacterial Compounds

Extraction of antibacterial compounds was carried out by modifying procedure reported by Ahmad and Ramadhan (2014). Dayak onions in powder form were extracted using fractionation method with redistilled *n*-hexane, ethyl acetate, and ethanol 96% sequentially. Firstly, powder Dayak onions were extracted with *n*-hexane (1:2 w/v) for 3 × 24 hours with occasional stirring. After three days, the mixture was filtered to give *n*-hexane filtrate and the residue-I. The *n*-hexane filtrate was evaporated using a rotary evaporator at 50°C and then concentrated to give *n*-hexane extract. Using the residue-I, the procedure was repeated with ethyl acetate to give ethyl acetate extract. Finally, the residue-II from ethyl acetate extraction was extracted with ethanol 96% to give ethanol extract. Each extract was weighed and stored in a dry and tightly closed container.

The preliminary phytochemical analysis of each extract was performed following the standard method (Harborne 1987). Each extract was qualitatively screened to identify the presence of various active compounds like flavonoid, alkaloid, saponin, triterpenoid, steroid and tannin.

### Determination of Antibacterial Activity

Antibacterial activity of each extract (hexane, ethyl acetate, ethanol 96% extracts) was determined by disc diffusion method or Kirby-Bauer method (Bauer *et al.* 1966). Each extract consisted of five different concentrations (2, 4, 6, 8 and 10 mg/mL). The pathogenic bacteria used were MRSA, *B. cereus*, *Shigella* sp. and *P. aeruginosa*. The test bacteria used were obtained from the Laboratory of Microbiology, Universitas Jenderal Soedirman, Purwokerto Indonesia. Each anti-bacterial assay was carried out in four replicates. As a positive control was cefadroxil 30 μg/mL in sterile aquadest, and as a negative control was a sterile aquadest.

All test organisms were cultured in Nutrient Broth (Merck, Germany) for overnight at 37°C before being used in the antibacterial assay. Antibacterial assay was carried out as follow. Paper discs (ø 6 mm, thickness 0.5 mm) were impregnated with 15 μL of each extracts solution with specific concentrations, air dried, and put on agar plates inoculated with test bacteria. The plates were incubated at 37°C for 24 h. Then, the diameter of the inhibition zone was measured to the nearest millimeter (mm). The inhibition activity was calculated by the equation (Ashshobirin *et al.* 2014):

$$\text{Inhibition activity }(\%)=\frac{\left(d_{2}-d_{1}\right)}{d_{1}}\times 100\%$$

where *d*_1_ = diameter of paper disc (6 mm) and *d*_2_ = diameter of inhibition zone (mm).

All data were analysed using analysis of variance (ANOVA). The results from ANOVA analysis which showed significant diversity was analysed further with Duncan’s Multiple Range Test (DMRT) with a 95% confidence level (*α* = 0.05).

The lowest concentration of each extract capable of inhibiting the growth of test bacteria was recorded as the minimum inhibitory concentration (MIC).

Extracts with the highest antibacterial activity towards MRSA, *B. cereus*, *Shigella* sp., and *P. aeruginosa* have then calculated its antibacterial effectiveness by comparing the diameter of inhibition zone from extract with that of a positive control, antibiotic cefadroxil 30 μg/mL. The antibacterial effectiveness of extract to the antibiotic was calculated by the equation (Orho *et al.* 2015):

$$\text{Antibacterial effectiveness}=\frac{D}{D_{a}}\times 100\%$$

where, *D* = diameter of inhibition zone from extract (mm) and *D_a_* = diameter of inhibition zone from positive control (mm).

### Assignment of Antibacterial Compounds

Extract with the highest antibacterial activity was assigned via Thin Layer Chromatography (TLC) on silica gel GF254 plate. The extract was eluted with chloroform, and the elution profile was detected under UV light (254 nm and 366 nm). Furthermore, an efficient technique namely Bioautography was also employed (Oedjijono 1992). The developed TLC-plate was put on test agar inoculated with the most inhibited bacteria tested. The plate agar was incubated at 37°C for 2 × 24 h, and then the inhibition zone was measured. The corresponding *R_f_* value was compared to a parallel developed TLC plate.

## RESULTS AND DISCUSSION

### Plant Identification

**Figure 1:**
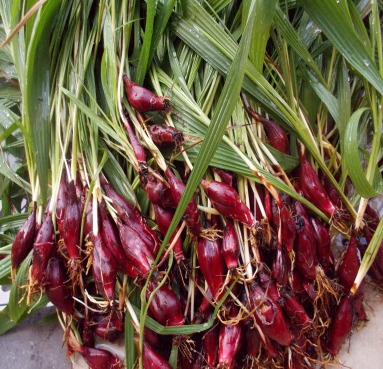

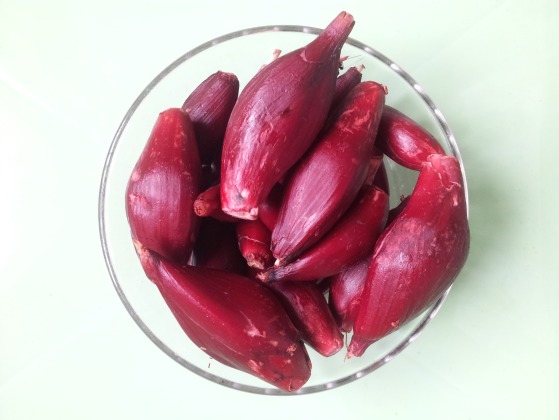

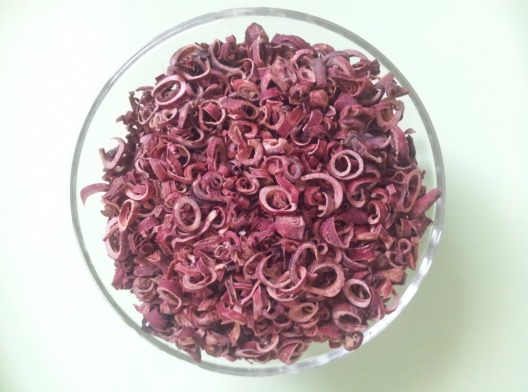
Dayak onion (a) the whole plants, (b) the bright-red bulbs, and (c) the dried slices of the bulbs.

Dayak onion is an annual herb plant with 30–40 cm of height. The leaves are green, single, pointed like ribbons with flat or un-serrated edges. It has tiny white flowers and bright-red bulb resembling of red onions (Fig. 1). The results of plant identification certified the sample (191/FB.Unsoed/TaksTumb/XI/2015) as follow:

**Table:** 

Family	:	Iridaceae
Specimen	:	*Sisyrinchium palmifolium* L.
Synonym	:	*Eleutherine palmifolia* (L.) Merr
Local name	:	Bawang Dayak (Dayak onion)
Reference	:	Mant. Pl. 1: 122 (1767)

### Extraction of Antibacterial Compound

The fresh bulb of onion was sliced and dried to reduce water content. The dried slices were grounded to produce a fine powder with the aim of expanding the surface for the extraction process to occur effectively. The yield obtained was 15%, based on the comparison between the weight of the resulting dried sample and the weight of fresh sample used.

The extraction process was performed gradually using three different polarities of solvents. In doing so, the bioactive compounds could be extracted based on its polarity level to maximise the extraction process. The use of *n*-hexane solvent was aimed to extract nonpolar compounds; ethyl acetate solvent to extract the semi-polar compound, and ethanol 96% solvent to extract the polar compound. After extraction, the solvent was evaporated to give a dried extract. The yield of dried extract for each solvent is presented in Table 1. Based on the extraction yield, it was apparent that the bulb of Dayak onion mostly contained polar compounds (1.85%) compare to semi-polar compounds (1.41%) and non-polar compounds (0.67%).

The qualitative phytochemical analysis was done to identify chemical components in each extract. The results of preliminary phytochemical assay indicated that Dayak bulb contained flavonoid, alkaloid, triterpenoid, steroid and tannin (Table 2). Based on the qualitative tests, ethanol extract positively indicated the presence of flavonoid, triterpenoid, and tannin. Ethyl acetate extract showed positive results indicated the presence of alkaloid and steroid; whereas *n*-hexane extract gave a positive result for steroid only. The chemical compounds contained in each extract most likely would act as antibacterial with a different mechanism.

Flavonoid is the largest group of phenol compounds that effectively inhibit the growth of viruses, bacteria, and fungi. Flavonoids inhibit the growth of bacteria by inhibiting the synthesis of nucleic acid, the function of the cell membrane, and energy metabolism (Cushnie & Lamb 2005). Alkaloids act as an antibacterial by inhibiting the synthesis of the cell which causes lysis of the cells (Lamothe *et al.* 2009). Triterpenoid as an antibacterial reacts with the porin (trans-membrane protein) in the outer membrane of the bacterial cell wall and forms a strong polymer bond resulting in the destruction of the porine. Damage to the porine will reduce the permeability of bacterial cell walls, decrease the nutrients uptake, and inhibit bacterial growth (Cowan 1999). Steroids can interact with phospholipid cell membranes that are permeable to lipophilic compounds that decrease membrane integrity as well as the morphology of cell membranes. Cells become brittle and lysis, eventually (Ahmed 2007). The mechanism of action of tannin as an antibacterial is by contracting the cell wall or cell membrane, thus disrupting the permeability of the cell itself. Due to disruption of the permeability, the cell cannot perform life activities so that its growth is hampered or even dead (Ajizah 2004).

### Antibacterial Activity of Extracts

**Figure 2:**
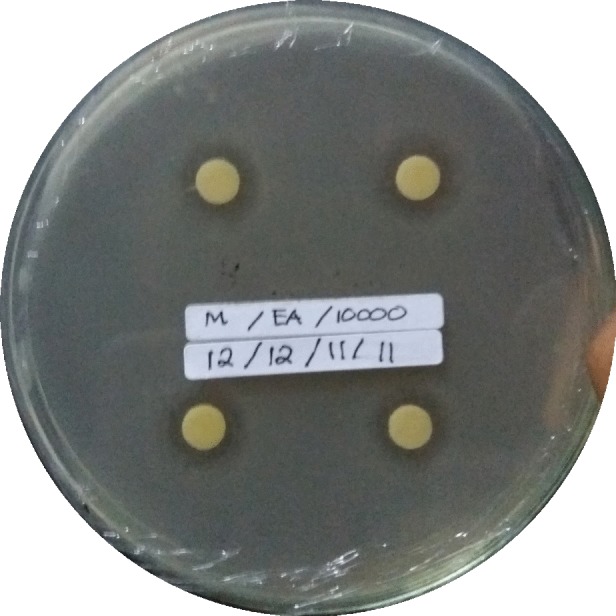

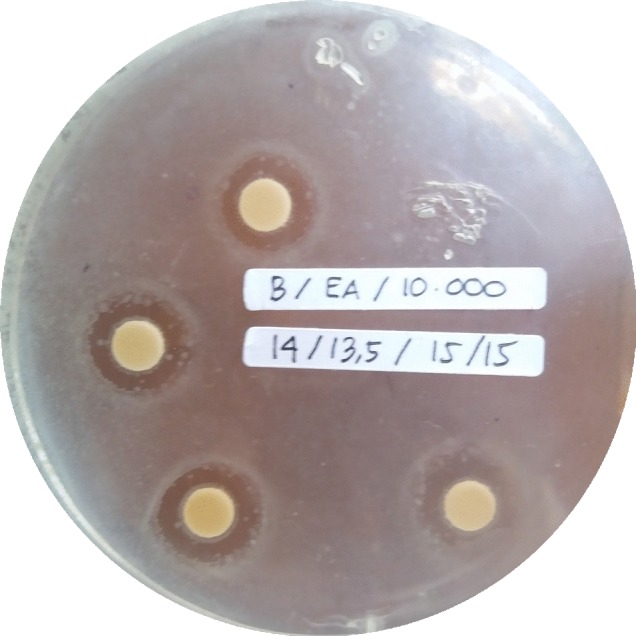

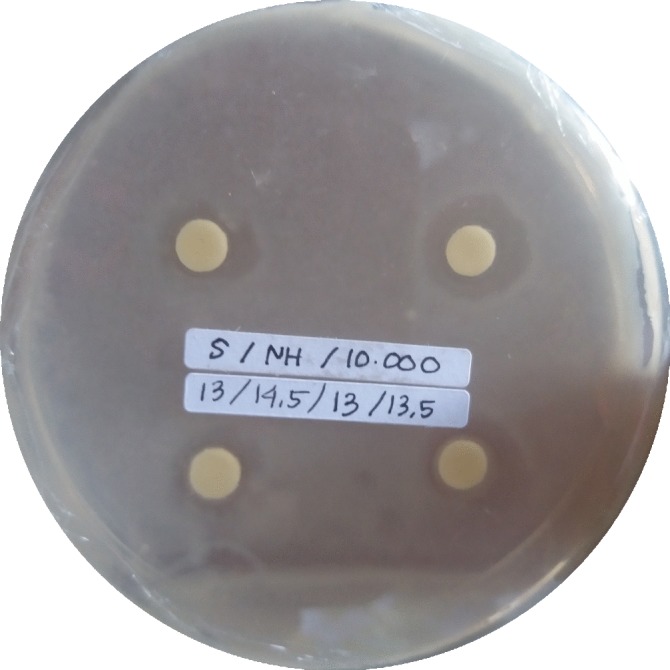

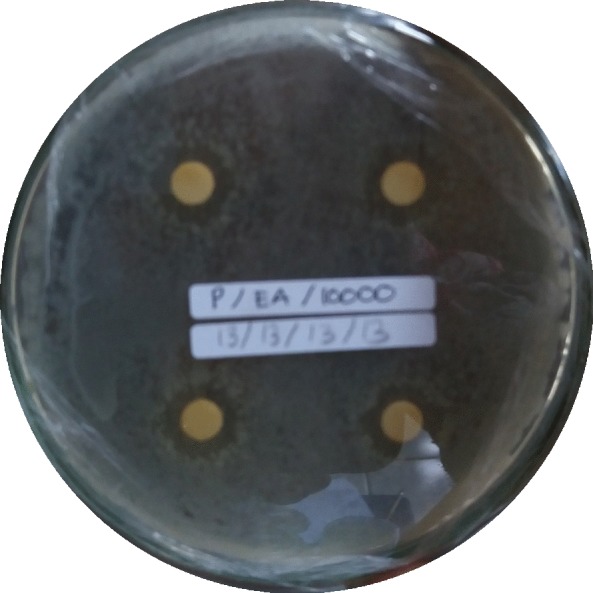
Inhibition zone from antibacterial activity assay (a) ethyl acetate extract (10 mg/mL) against MRSA, (b) ethyl acetate extract (10 mg/mL) against *B. cereus,* (c) hexane extract (10 mg/mL) against *Shigella* sp., and (d) ethyl acetate extract (10 mg/mL) against *P. aeruginosa.*

**Figure 3:**
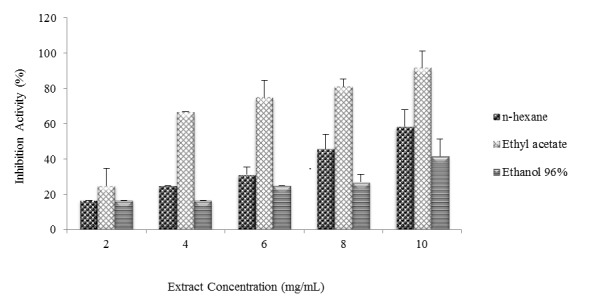

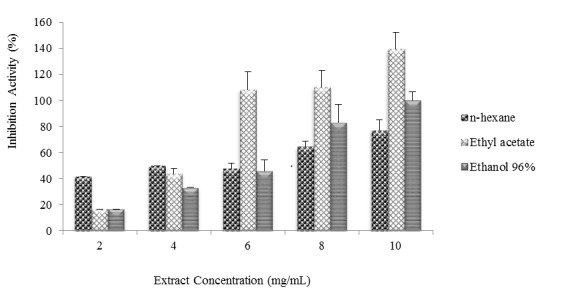

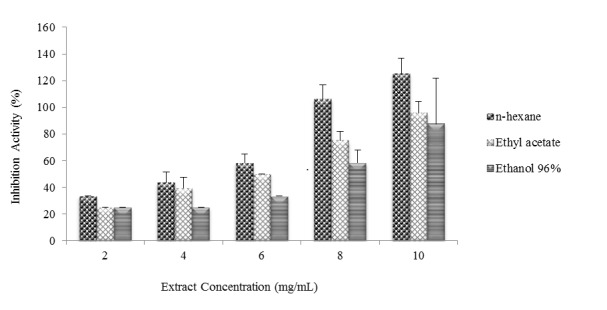

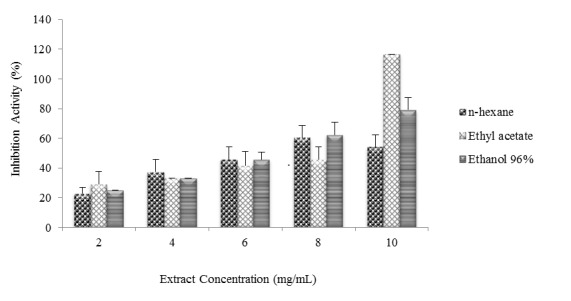
The profile of inhibition activities (%) from n-hexane, ethyl acetate, and ethanol 96% extracts against the growth of pathogenic bacteria (a) MRSA, (b) *B. cereus,* (c) *Shigella* sp., and (d) *P. aeruginosa.* The concentration of each extract used were 2, 4, 6, 8, and 10 mg/mL. The higher concentration used gave higher inhibition activity. The highest inhibition activity (139.58%) was 10 mg/mL.

The results from antibacterial activity test indicated that *n*-hexane, ethyl acetate, and ethanol 96% extracts positively inhibit the growth of MRSA, *B. cereus*, *Shigella* sp., and *P. aeruginosa* (Fig. 2). Different type of extracts and different concentration gave different inhibition activities. Increasing the concentrations of all extract was directly correlated with the increasing inhibition activities. Thus, the higher the concentration of extract used, the higher the inhibition activity (Fig. 3). Increasing concentrations would result in the higher composition of bioactive compounds in the extract, so the ability to inhibit bacterial growth was also getting stronger.

The results from analysis of variance showed that the type of extracts, the variation of extract concentrations, and the interaction between the two significantly inhibited the growth of MRSA, *B. cereus*, *Shigella* sp. and *P. aeruginosa* inhibition at 95% confidence level (*α* = 0.05). Further Duncan tests indicated that the interaction between the extracts and concentration of 10 mg/mL gave the highest inhibition activity. The highest inhibition activity against MRSA was ethyl acetate extract (10 mg/mL, 91.67% inhibition, Fig. 3a); against *B. cereus* was ethyl acetate extract (10 mg/mL, 139.58% inhibition, Fig. 3b); against *Shigella* sp. was *n*-hexane extract (10 mg/mL, 125% inhibition, Fig. 3c); and against *P. aeruginosa* was ethyl acetate extract (10 mg/mL, 116.67% inhibition, Fig. 3d). These findings were summarised in Table 3.

The antibacterial effectiveness was then calculated using antibiotic cefadroxil as a positive control. The research results showed that the antibacterial effectiveness of 10 mg/mL of ethyl acetate extract against cefadroxil antibiotics 30 μg/mL on the growth of *P. aeruginosa* and *B. cereus* was higher than that of *Shigella* sp. and MRSA. This result suggested that ethyl acetate extract was more effective in inhibiting the growth of *P. aeruginosa* and *B. cereus* compared to 30μg/mL cefadroxil antibiotics (Table 3).

Based on the antibacterial activity tests, the ethyl acetate extract of Dayak onion gave significant effect in inhibiting most bacteria tested. Probably, the extracted bioactive compounds affect bacterial cell walls which are known to have two solubility properties: hydrophilic and lipophilic. The semi-polar compounds in ethyl acetate extract were thought to have a higher affinity so that they could interact better with the cell wall. The hydrophilic properties are necessary to ensure the soluble compounds in the water phase which are the microbial life spans, and the compounds are acting on the hydrophobic cell membrane require lipophilic properties. This explanation was in accordance with the statement of Kanazawa *et al.* (1995) which stated that a compound having an optimum affinity would have an optimum antimicrobial affinity because antimicrobial compounds require hydrophilic-hydrophobic balance in their interactions with bacteria.

Phytochemical analysis indicated the presence of alkaloids in ethyl acetate extract. The alkaloids are known to have potential as antibacterial because they can damage the cell wall. Lamothe *et al.* (2009) stated that the alkaloids compound served as antibacterial by inhibition of cell wall synthesis which can cause lysis of the cell, and eventually lead to the cell death. Harborne (1987) stated that alkaloids could interfere with the formation of peptidoglycan components in bacterial cells, thus causing the loss of cell wall function as an osmotic pressure protector. The lack of peptidoglycan components causes the bacterial cell to become sensitive to osmotic pressure, and the high osmotic pressure in the bacterial cell will cause lysis of the bacterial cell. The highest inhibition activity was obtained from ethyl acetate extract against *B. cereus* (139.58%) which indicated that *B. cereus* was the most sensitive bacteria towards Dayak onion than other bacteria tested. The inhibition activity toward Gram-positive bacteria (*B. cereus*) was higher than Gram-negative bacteria (*Shigella* sp. and *P. aeruginosa*). This observation was in agreement that Gram-positive bacteria tend to be more sensitive to antibacterial compounds than Gram-negative bacteria (Radji 2009). This is due to the differences in the structure of bacterial cell wall. In particular, the cell wall of Gram-positive bacteria is simpler than Gram-negative cell wall bacteria. Grampositive bacteria have cell walls consisting of peptidoglycan layers with thick and rigid structures. Oppositely, Gram-negative bacteria have thinner cell walls, consisting of one or more peptidoglycan layers and high lipid content (Pelczar & Chan 1986). Another perception is that the permeability of the outermost membrane of the bacterial cell wall is determined by the presence of protein molecules in the form of porin (Radji 2009). Porins contained in the outer membrane is thought to hamper bioactive molecules entering bacterial cells. This is due to the difference in properties between the porin and the components contained in the extract, in which the porin is nonpolar, and the Dayak bulb extract is more polar. The difference in properties probably caused the antibacterial compounds in Dayak onion extracts was more difficult to enter the bacterial cells of *Shigella* sp and *P. aeruginosa* (Gram-negatives), so the resulting inhibition zone was smaller than that of *B. cereus* (Gram-positive).

It is interesting to note that MRSA could be affected by ethyl acetate extract of Dayak onion with inhibition activity of 91.67%. MRSA is a *Staphylococcus aureus* which resistant to antibiotic Methicillin. The pathogenicity of MRSA is higher than that of *S. aureus*, due to its resistance which causes the severity of the disease because of MRSA infections. This particular result emphasised the important of Dayak onion as an alternative antibacterial to deal with microorganisms that have been resistant to commercial antibiotics.

**Figure 4:**
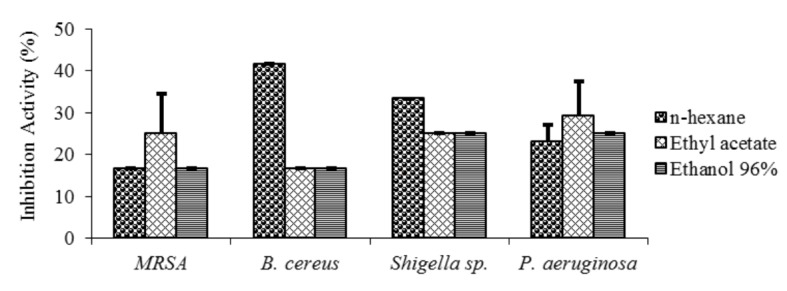
The inhibition activity (%) at MIC (2 mg/mL) of hexane, ethyl acetate, and ethanol 96% extracts against MRSA, *B. cereus*, *Shigella* sp., and *P. aeruginosa*. MRSA bacteria showed significant differences in inhibition activity with other bacteria.

The minimum inhibitory concentration (MIC) of the extract was determined to know the minimum concentration required to inhibit or kill bacterial growth. The results showed that the minimum concentration of Dayak onion extracts against all bacteria tested was 2 mg/mL. The inhibition activity (%) at MIC (2 mg/mL) of hexane, ethyl acetate, and ethanol 96% extracts against MRSA, *B. cereus*, *Shigella* sp. and *P. aeruginosa* is shown in Fig. 4. The result of variance analysis indicated that the type of extracts, the type of test bacteria, and the interaction between the two with the minimum inhibitory concentration had a significant effect (*p* > 0.05) on the inhibition activity. The 5% DMRT test results showed a marked difference to each type of extracts. The 5% DMRT test against the mean of inhibition activity for bacteria of *B. cereus*, *Shigella* sp. and *P. aeruginosa* at a concentration of 2 mg/mL showed no significant difference. This means that those three bacteria have the same susceptibility to the inhibition process using Dayak bulb extracts at a concentration of 2 mg/mL. On the contrary, MRSA bacteria showed significant differences with other bacteria. This means MRSA has a different susceptibility to the inhibition process using Dayak bulb extracts at a concentration of 2 mg/mL.

### Assignment of Antibacterial Compounds

**Figure 5:**
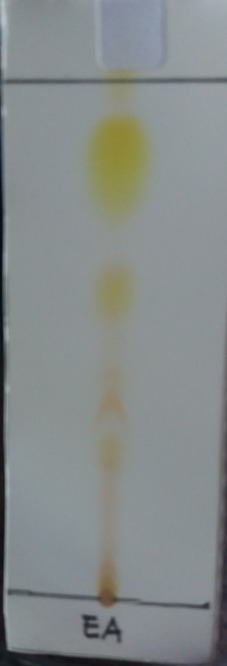

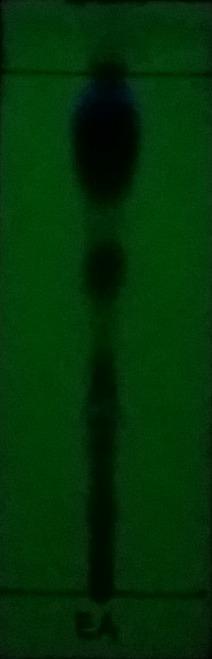

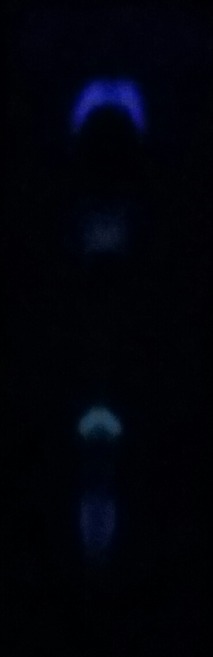
TLC chromatogram of ethyl acetate extract with silica gel GF254 as the stationary phase and chloroform as the mobile phase. (a) direct observation; (b) observation at λ254, and (c) observation at λ366 nm. The results showed four spots with different *R_f_*.

Based on the bacterial inhibition assay, it was found that ethyl acetate extract has the highest inhibition activity against *B. cereus* with inhibition activity 139.58%. Accordingly, ethyl acetate extract was further analysed by thin layer chromatography (TLC) to preliminary separate the compounds. The results from TLC showed four spots at 254 nm wavelength and four spots at 366 nm wavelength. The *R_f_* values were 0.29, 0.42, 0.62, and 0.84 at 254 wavelengths; whereas the *R_f_* values at the 366 nm wavelength were 0.18, 0.36, 0.67, and 0.90 (Fig. 5).

**Figure 6:**
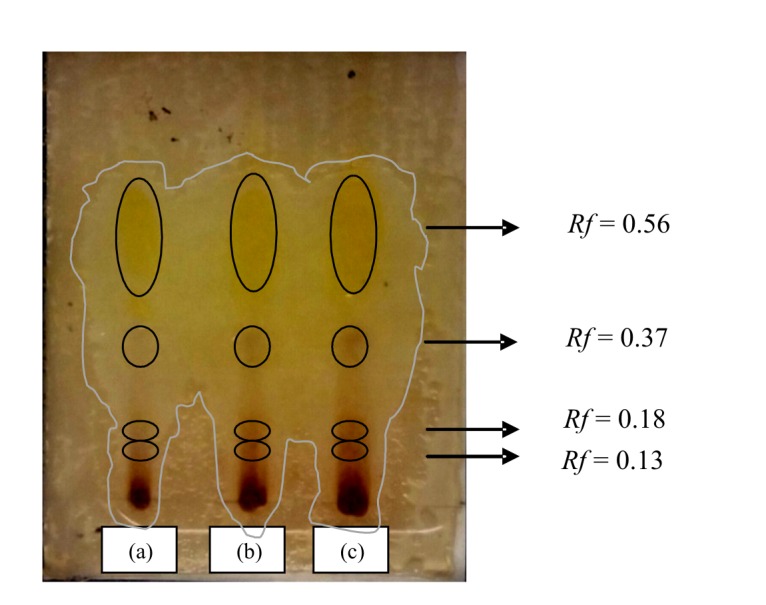
Bioautography of ethyl acetate extract against *B. cereus* with varying amounts of samples dropped on the TLC plate, (a) 3 mL, (b) 6 mL, and (c) 9 mL of ethyl acetate extract. The formation of clear zone indicated the inhibition activity of ethyl acetate extract against *B. cereus*. The highest inhibition activity was observed from a compound with *R_f_* of 0.56.

After TLC, the four spots obtained were further analysed with a bioautography method to identify which spot that has antibacterial activity against *B. cereus*. The bioautography result showed that the ethyl acetate extract could inhibit the growth of bacteria *B. cereus*, which was characterised by the formation of clear zone in the spots on the chromatogram plate. It could be seen that the extract contained four types of active compounds with *R_f_* values of 0.13 (spot 1), 0.18 (spot 2), 0.37 (spot 3), and 0.56 (spot 4) respectively. It was observed that spots 3 and 4 have larger inhibition zone than spots 1 and 2. This might indicate that the compounds with *R_f_* values of 0.37 and 0.56 have higher inhibition activity against *B. cereus* than compounds with *R_f_* values of 0.18 and 0.13 (Fig. 6).

## CONCLUSION

Based on phytochemical screening, secondary metabolite present in the *n-*hexane extract was steroids, in ethyl acetate extract were alkaloids and steroids, and in ethanol 96% extract were flavonoids, triterpenoids, and tannins. Extracts *n*-hexane, ethyl acetate, and ethanol 96% of Dayak onion have antibacterial activity against pathogenic bacteria, specifically MRSA, *B. cereus*, *Shigella* sp., and *P. aeruginosa* with the highest inhibition activity was achieved at a concentration of 10 mg/mL. The minimum inhibition concentration of the extracts against MRSA, *B. cereus*, *Shigella* sp. and *P. aeruginosa* was 2 mg/mL. The highest inhibition activity was obtained from ethyl acetate extract against *B. cereus* with 139.58% of inhibition activity. Ethyl acetate extract gave four spots in TLC eluted with 100% chloroform. The *R_f_* values were 0.29, 0.42, 0.62, 0.84 at λ254 nm; and 0.18, 0.36, 0.67, 0.90 at λ366 nm. Bioautography results showed that all four spots have antibacterial activity against *B. cereus*. Compounds with *R_f_* values of 0.37 and 0.56 have higher bacterial inhibition activity than compounds with *R_f_* values of 0.18 and 0.13.

## Figures and Tables

**Table 1:** The yields of dried extract

Solvents	Extract (gram)	Yield (%)
*n-*hexane	6.03	0.67
Ethyl Acetate	12.73	1.41
Ethanol 96%	16.67	1.85
Total Yield		3.93

**Table 2:** The results of qualitative phytochemical screening

Chemical component	Extract		

	*n-*hexane	Ethyl acetate	Ethanol 96%
Flavonoid	−	−	+
Alkaloid	−	+	−
Saponin	−	−	−
Triterpenoid	−	−	+
Steroid	+	+	−
Tannin	−	−	+

*Notes*: + = presence of chemical component; − = absence of chemical component

**Table 3:** Antibacterial effectiveness of the extract with the highest inhibition activity towards tested bacteria

Test bacteria	Extract	Extract conc. (mg/mL)	Inhibition activity (%)	Antibacterial effectiveness (%)*
MRSA	Ethyl acetate	10	91.67	76.67
*B. cereus*	Ethyl acetate	10	139.58	110.61
*Shigella* sp.	*n-*hexane	10	125.00	79.41
*P. aeruginosa*	Ethyl acetate	10	116.67	162.50

* positive control: cefadroxil 30 μg/mL
